# Crystal structures of *Mycobacterium tuberculosis* HspAT and ArAT reveal structural basis of their distinct substrate specificities

**DOI:** 10.1038/srep18880

**Published:** 2016-01-07

**Authors:** Nazia Nasir, Avishek Anant, Rajan Vyas, Bichitra Kumar Biswal

**Affiliations:** 1 Protein Crystallography Laboratory, National Institute of Immunology, Aruna Asaf Ali Marg, New Delhi, Delhi, 110067, India

## Abstract

Aminotransferases of subfamily Iβ, which include histidinol phosphate aminotransferases (HspATs) and aromatic amino acid aminotransferases (ArATs), are structurally similar but possess distinct substrate specificities. This study, encompassing structural and biochemical characterisation of HspAT and ArAT from *Mycobacterium tuberculosis* demonstrates that the residues lining the substrate binding pocket and N-terminal lid are the primary determinants of their substrate specificities. In *m*HspAT, hydrophilic residues in the substrate binding pocket and N-terminal lid allow the entry and binding of its preferential substrate, Hsp. On the other hand, the hydrophobic nature of both the substrate binding pocket and the N-terminal lid of *m*ArAT is responsible for the discrimination of a polar substrate such as Hsp, while facilitating the binding of Phe and other aromatic residues such as Tyr and Trp. In addition, the present study delineates the ligand induced conformational rearrangements, providing insights into the plasticity of aminotransferases. Furthermore, the study also demonstrates that the adventitiously bound ligand 2-(N-morpholino)ethanesulfonic acid (MES) is indeed a specific inhibitor of HspAT. These results suggest that previously untapped morpholine-ring scaffold compounds could be explored for the design of new anti-TB agents.

Aminotransferases (ATs) catalyze reactions in amino acid biosynthesis and other metabolic pathways in almost all organisms ranging from prokaryotes to eukaryotes. These enzymes show dexterity in either being exclusive for a particular pathway or in having multiple catalytic capabilities with functional overlaps between various cellular processes^1^. They belong to the superfamily I of PLP (pyridoxal 5′-phosphate)-dependent enzymes^2^ and exhibit a characteristic ping pong bi-bi mechanism. The first half of the reaction involves conversion of PLP into pyridoxamine 5′-phosphate (PMP)^3^. The second half of the reaction mostly entails the recognition and catalysis of α- amino acids as substrates^3^. However histidinol phosphate aminotransferases (HspATs) specifically recognize an amino-organo phosphate, histidinol phosphate (Hsp). This substrate has an anionic group which is different in size, shape, and charge^4^ from that of α- amino acids. HspATs belong to the subfamily Iβ of aminotransferases, which also includes aromatic amino acid aminotransferases (ArATs)^2^. While the former enzymes catalyze the α- elimination and replacement of an amino group from L-glutamate onto Hsp, a precursor of L-histidine (His)^5^, the latter preferentialy utilize aromatic amino acids Phe, Tyr and/or Trp as their substrates (Fig. 1). Both the 1β aminotransferases from other organisms have been reported to display the characteristic broad specificity with cross-over functions between two independent pathways^6^. However, the factors responsible for the differential substrate recognition by these enzymes are yet to be fully explored.

In order to decipher the structural basis of their substrate specificities and to provide a rational, we structurally and biochemically characterized two 1β aminotransferases from *Mycobacterium tuberculosis* (*Mtb*), an HspAT, encoded by *rv1600* (mHspAT) and a putative phenylalanine aminotransferase, encoded by *rv3772*. The latter enzyme was earlier annotated as HspAT suggesting its involvement in His pathway^7^. However, experimental characterisation of this aminotransferase in the current study clearly demonstrates high catalytic preference for Phe, Tyr and Trp instead for Hsp. Therefore, we propose its reannotation of as an aromatic amino acid aminotransferase (*m*ArAT). Through systematic kinetic analysis we calculated the catalytic efficiencies of the two enzymes for their respective substrates. Our structural studies encompassing the crystal structures of native and ligand bound *m*HspAT as well as two ligand bound structures of *m*ArAT demonstrate that residues lining the substrate binding pocket and the N-terminal lid dictate the distinct substrate preferences of these two mycobacterial aminotransferases. In addition, the present study also shows that MES is a competitive inhibitor of *m*HspAT, opening up a new prospect for the design of morpholine-ring derived anti-TB compounds.

## Results

### Kinetic analysis of *m*HspAT and *m*ArAT

The transamination activity of *m*HspAT and *m*ArAT were evaluated using steady state kinetics in a two-step aminotransferase assay (Supplementary Fig. S1). *m*HspAT exhibited excellent transaminase activity for its known substrate Hsp, whereas *m*ArAT showed no significant activity for the same. *m*ArAT was assayed for its affinity for Phe and other naturally occurring L-amino acids. Notably, it showed excellent activity not only for Phe, but also for Tyr and Trp. Similarly, *m*HspAT was tested for its broad specificity towards other L-amino acids (Supplementary Fig. S2). The relative specific activities with the array of substrates clearly demonstrated activity of *m*HspAT for Hsp as well as moderate efficacy for aromatic amino acids. *m*ArAT was more specific towards the aromatic amino acids. The catalytic rates of *m*HspAT for Hsp, Phe and Tyr and of *m*ArAT for Phe, Tyr and Trp were measured at varying concentrations of substrates and the results conformed to the standard Michaelis-Menten kinetics (Table 1). The values for the catalytic efficiencies of *m*HspAT for Hsp are relatively higher compared to counterparts from *Corynebacterium glutamicum* (*C. glutamicum*)^8^ and the thermophile *Thermotoga maritima* (*T. maritima*)^9^ (Supplementary Table S1). On the other hand, the homolog from *Bacillus subtilis* (*b*HspAT) shows almost three-times higher affinity for Hsp, and also exhibits much higher affinities for Phe and Tyr as compared to the *m*HspAT^10^. The trend for catalytic efficiency (*k*_*cat*_*/K*_*M*_) of *m*HspAT for its substrates was observed as Hsp > Tyr > Phe while that for *m*ArAT was Phe > Tyr > Trp.

### *m*HspAT and *m*ArAT exhibit topology similar to homologues belonging to subfamily Iβ aminotransferases

**Figure 2:**
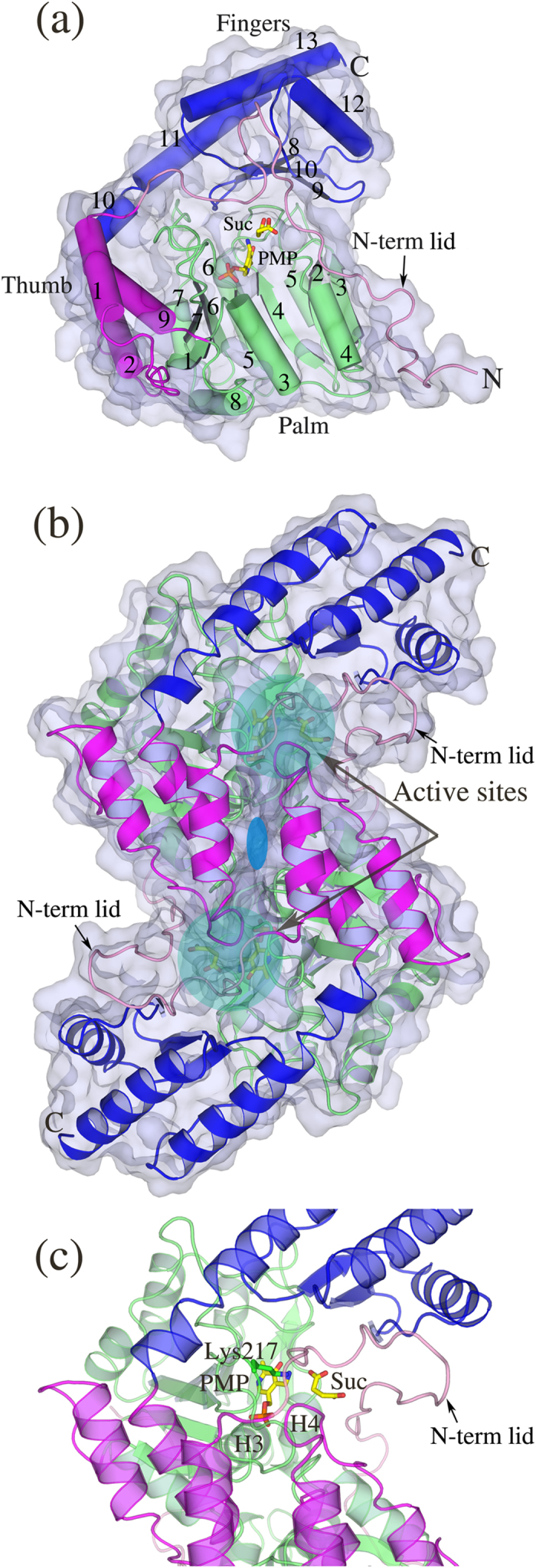
Representative 3D structures of *m*HspAT and *m*ArAT. (**a**) The domain structures of *m*ArAT monomer bound to PMP and succinic acid (Suc) is shown where the N-terminal lid, palm, thumb and fingers domains are colored in pink, lime, magenta and blue respectively. *m*HspAT also adopts similar topology. (**b**) Both *m*ArAT-(shown) and *m*HspAT-ligand bound forms adopt the conserved dimeric structure. The two-fold symmetry that relates the monomers of dimer is represented by the blue ellipse. (**c**) Zoom in view of *m*ArAT active site region. Shown in stick model are the catalytic lysine and the PMP and succinate molecules.

To decipher the structural basis of distinct substrate specificities of *m*HspAT and *m*ArAT, we elucidated their three-dimensional (3D) structures. The apo and liganded forms of *m*HspAT crystallized in hexagonal and orthorhombic space groups, respectively, whereas, both *m*ArAT-succinate and *m*ArAT-Phe complexes crystallized in orthorhombic space groups. Both the class of enzymes display the canonical aminotransferase fold of the PLP-dependent Iβ subfamily. Their tertiary structure can be described as analogous to a curved left hand with clustering of three distinct structural motifs in the palm, thumb and fingers positions (Fig. 2a). The PLP-binding domain in the palm position (palm domain) encompasses a major portion of the polypeptide chain and consists of a seven-stranded β-sheet sandwiched between two bundles, each with three α- helices. The C-terminal domain resembling the fingers (fingers domain) forms a roof-shaped three-helix bundle resting over a small β-sheet. Helix 9 of the thumb domain connects the palm and fingers domains. An N-terminal ‘lid’, consisting of an approximately 40-residue long loop, protrudes from the thumb domain and closes over the PLP-binding domain (Fig. 2a).

Typically, the biologically active form of Iβ aminotransferases is a homodimer whose monomers are related by a pseudo 2-fold symmetry. Both *m*HspAT and *m*ArAT adopt the similar functional unit whose protomers are aligned in an inverted manner with the axis of the molecular dyad passing through the interface (Fig. 2b). Residues that form the interface of the dimer protrude from the palm and thumb domains as well as the N-terminal lid of both protomers. Both the dimers of *m*HpsAT and *m*ArAT contain two active sites in similar positions approximately 25 Å apart, with residues from both chains lining the cleft (Fig. 2b,c). The active site cavity is lined by residues mainly contributed by the palm domain. The fingers domain forms the roof of the cleft and the N-terminal lid shields the active side from the solvent in the ligand bound form. The crystallographic dimerization involves the burial of approximately a fifth of the surface area (17,000 Å^2^) of an individual monomer (Supplementary Table S2). The two structures align to each other with a root mean square deviation (rmsd) of 2.25 Å over 543 Cα atom pairs.

### Cofactor binding triggers large conformational changes in *m*HspAT

**Figure 3:**
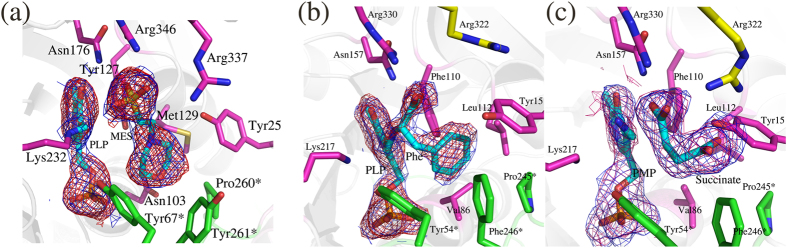
Ligand recognition by *m*HspAT and *m*ArAT. (**a**) The electron density (ED) maps (2|F_o_|–|F_c_| (blue) and |F_o_|–|F_c_| (red) at 1σ and 2.5σ contour levels respectively) for the holo form shows MES bound near PLP. ED maps (2|F_o_|–|F_c_| (blue) and |F_o_|–|F_c_| (red) at 1σ and 2.5σ contour levels, respectively are shown for PLP-Phe (**b**) and PMP and succinate molecules (**c**) bound in the active site of *m*ArAT complexes. The two distinct substrates are recognized by *m*ArAT using the “arginine switch” mechanism, in which Arg322 (yellow) moves away from the active site to accommodate the neutral phenyl ring of Phe.

**Figure 4:**
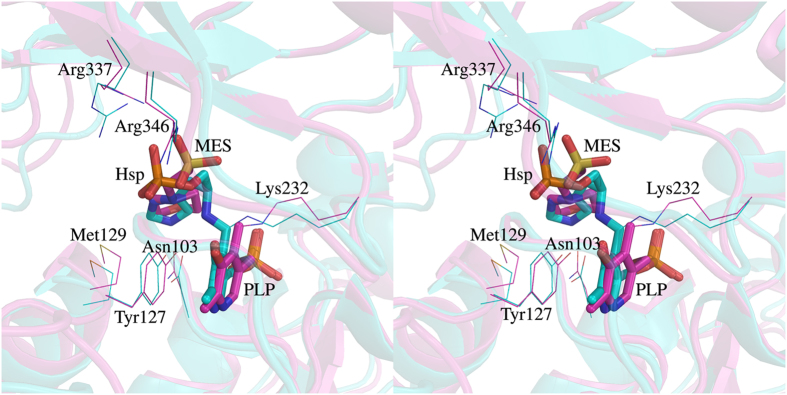
Comparison of the active site regions of *Mtb* and *E. coli* HspATs. Structural differences between *Mtb* (magenta) and *E. coli* (cyan; P. D. B. ID: 1FG3) HspATs are presented in stereo mode. Both the structures were superimposed with 1.6 Å rmsd (over 639 Cα atom pairs). The active site residues in the both the structures are mostly conserved and MES in *m*HspAT binds in a manner similar to Hsp in *e*HspAT. The morphiline-ring of MES and the imidazole ring of Hsp overlap and their respective sulphate and phosphate groups are also positioned similarly. The PLP binding in both cases is almost identical. The labeled residues are with respect to *m*HspAT.

**Figure 5:**
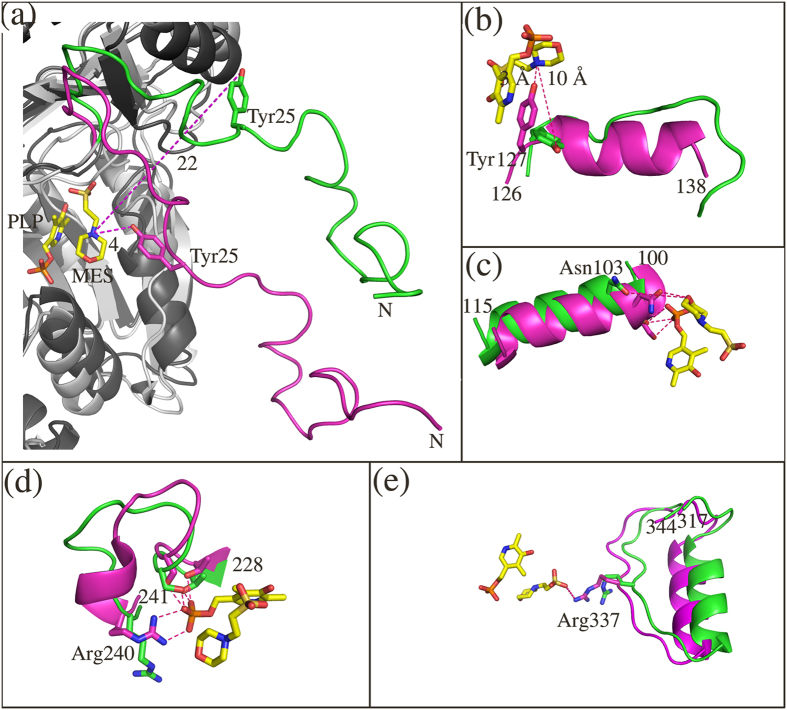
Mapping the conformational changes in the vicinity of active site region of *m*HspAT upon ligand binding. (**a**) The N-terminal lid adopts a closed conformation upon ligand binding, bringing in Tyr25. The ligand free and MES-bound structures are shown in green and magenta respectively. (**b**) Remodelling of a loop region to an ordered helix flips in the active site Tyr127 which interacts through its hydroxyl group with the nitrogen of the morpholine-ring of MES. (**c**) Concerted displacement of helix 3 results in the movement of active site Asn103 whose hydroxyl group interacts with the ring oxygen of MES. (**d**) The binding of the ligands in the active site also leads to the helicalization of a small loop region which flips in Arg240 which interacts with the phosphate tail of PLP. Further, Thr229 and Ser221 move away from the active site to prevent steric clashes when the cofactor binds in the active site pocket. (**e**) The inward movement of a helix of the C-terminal domain brings in Arg337 to an ideal position which provides binding energy for the MES stabilization making hydrogen bond interaction with its O3S atom.

Analyses of the crystal structures of the holo form of *m*HspAT showed the binding of an Hsp-like molecule, 2-(N-morpholino)ethanesulfonic acid (MES) (Supplementary Figs S3a and S3b), in the active site of the enzyme (Fig. 3a). Also observed was the binding of the PLP as an internal aldimine via a Schiff’s base with Lys232 Nζ. The orientation of the morpholine-ring compound is similar to that of Hsp in substrate bound *E. coli* HspAT (*e*HspAT; 31% sequence identity; Fig. 4)^11^,^12^ suggesting that MES binds in the active site of *m*HspAT. Superimposed structures of native and ligand-bound forms of *m*HspAT lucidly project various differences in the regions of the palm, thumb and fingers domains. It also reveals that the N-terminal lid is restructured upon binding of ligand (Fig. 5a–e), leading to a ‘closed’ conformation of the enzyme necessary for the binding and probable catalysis of the substrate. The lid pivots about Arg35, with an rmsd between its ‘open’ and ‘closed’ forms of 4.25 Å (over 41 Cα atom pairs) (Fig. 5a). The closing of the lid upon ligand binding causes Tyr25 to sweep into the active site region and interact with the ligand. The backbone of the C-terminal domain undergoes a subtle lateral shift (rmsd of 0.28 Å over 78 Cα aligned atoms) with only three residues undergoing conformational changes in their side chains. This is unlike the previously reported homologous structures where the C-terminal domain residues undergo major conformational changes upon transition between ‘open’ and ‘closed’ conformations^13^,^14^,^15^,^16^. In *m*HspAT, Arg337, Arg346 and Val339 in the C-terminal domain undergo a change in the conformation of their side chains. The conformational changes of these two Arg residues are also implicated in ligand binding, as described later.

The ‘open’ and ‘closed’ conformations of *m*HspAT monomers also impact the packing of the homodimer. The apo dimeric form of the enzyme adopts a relatively compact organization of the monomers compared to the typical dimeric arrangement seen for liganded form of aminotransferases. This results in a shift of 6.5 Å between the centre of masses (CoMs) of the monomers of the apo and holo forms of *m*HspAT dimer (Supplementary Fig. S4). Furthermore, other regions in the vicinity of the active site that undergo significant conformational changes upon interaction with ligands include helix formation in two loop regions and displacement of two helices (Fig. 5b–e). The two loop regions, Ser126-Thr138 and Phe236-Arg240, which become ordered to form helices result in the reorientation of Tyr127 and Arg240 which enables these residues to interact with the ligands (Fig. 5b,d). Concurrently, two helices (Fig. 5c,e) undergo concerted displacement, facilitating the interaction of the residues Asn103 and Arg337 with the ligands. Changes in the interactions of the residues at the interface are also observed, which include reshuffling of the four salt bridges between residues. The binding of the ligand thermodynamically stabilizes the protein as is exemplified by the increase in the number of H-bonds (from 20 to 31) and non-bonded interactions (from 306 to 390).

### Active site of *m*ArAT can accommodate a dicarboxylate and an aromatic amino acid

The binding of Phe and succinate (Fig. 3b,c) in the active site of *m*ArAT as observed in the crystal structures of *m*ArAT-PLP-Phe and *m*ArAT-PMP-succinate complexes revealed the molecular basis of substrate selectivity of *m*ArAT. The two liganded structures are virtually identical (an rmsd of 0.28 Å over 305 Cα atom pairs). In the substrate bound *m*ArAT complex, Phe is present in the active site of both the monomers of a homodimer, though it exists as a PLP-Phe external aldimine intermediate complex in one of the monomers, and as free-Phe in the other monomer. The carboxylate group of free-form of Phe is held in place through interactions with Asn157 and the PLP bound-form makes an additional interaction with Arg330. Utilizing the structural information gained from the two structures, we traced the plausible conformational changes that *m*ArAT undergoes - from accommodating a dicarboxylate to creating a suitable cavity for Phe entry and binding (Supplementary Movie S1). The N-terminal lid regulates the entry and binding of Phe by rearranging the side chains of Tyr15, along with Glu111, Leu112 and Arg322. A stretch of residues of the lid exhibit plasticity (Leu9 to Ala24, rmsd 2.9 Å over 16 aligned Cα pairs), by adopting different conformations in free- and Phe bound form. Tyr15 forms a H-bond with Arg322 when Phe is covalently linked to the cofactor in one monomer. In the other monomer, the carboxylate group of ‘free’ Phe form H-bond with Arg322. In the dicarboxylate bound form, succinate exists as a non-covalently linked moiety, forming a ‘twisted’ bidentate H-bond/ion pair with Arg322 and Arg330 of the C-terminal domain. Succinate essentially mimics L-glutamate, an amino donor (Supplementary Fig. S3c,d).

Rearrangement of H-bond network in ArAT from *Paracoccus denitrificans*^17^,^18^ and “arginine switch” in an engineered TyrAT from *E. coli*^19^, facilitate dual substrate recognition and binding. The present structures clearly demonstrate the role of the “arginine switch” mechanism in the binding of the carboxylate group of Phe in which Arg322 moves away from the active site to allow the access of the bulky head group of Phe (Fig. 3b,c). However, as our study suggests, the preferential binding of Phe and discrimination of Hsp in *m*ArAT is also impacted by the presence of specific active site residues.

### Mode of PLP binding is conserved in both *m*HspAT and *m*ArAT

**Figure 6:**
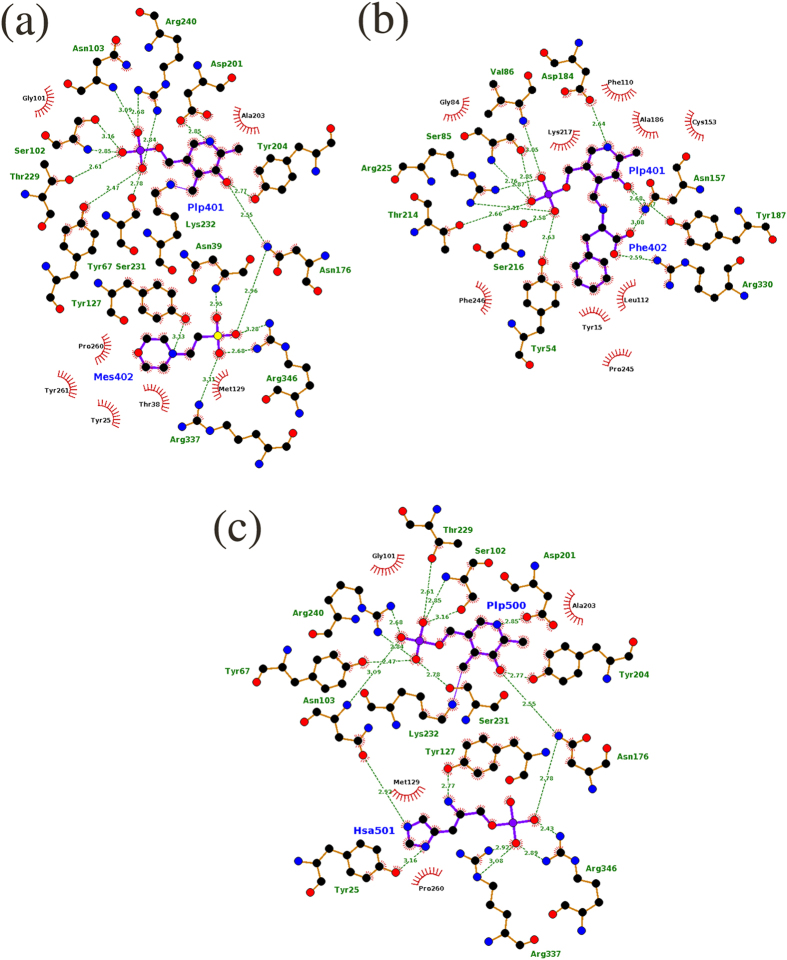
Schematic representations of the atomic interactions between (**a**) *m*HspAT and PLP-MES, (**b**) *m*ArAT and PLP-Phe and (**c**) *m*HspAT and PLP-Hsp (Hsa). In both enzymes, PLP makes stronger binding than the substrate. Residues which are involved in H-bond interaction (shown in green dotted lines with the corresponding donor-acceptor distance) are shown in ball and stick model, whereas those that are involved in van der Waals interactions with the ligands are shown in spikes. In panel (**a**), Tyr67, Pro260, and Tyr261 protrude from the adjacent molecule (chain B; PDB ID: 4R8D) of the dimer. Try54, Pro245 and Phe246, in panel (**b**), belong to the other molecule (chain D; PDB ID: 4R2N) of the dimer). In panel (**c**), Tyr67 and Pro260 protrude from the adjacent molecule.

The unifying factor amongst transaminases is the requirement of PLP as a cofactor. The cofactor PLP/PMP is lodged into the active sites of *m*HspAT and *m*ArAT through a number of interactions which are conserved across the family of Iβ aminotransferases^2^. In *m*HspAT, these include as many as nine H-bonds and a salt bridge formed between the active site Arg240 and the phosphate moiety of PLP (Fig. 6a). The pyridine ring of PLP bound in the active site of *m*HspAT is further stabilized by π-π stacking with Tyr127 and covalent linkage of its C4A atom with the Nζ of the active site Lys232. Similarly, H-bonds, a salt bridge and π-π stacking with the active site Phe110 stabilize the PLP in *m*ArAT (Fig. 6b). Since PLP-binding is largely conserved, the basis of substrate specificity of different classes of aminotransferases can only be attributed to the chemical environment of their substrate binding pocket.

### Hydropathicity of active site pockets dictates substrate specificities of *m*HspAT and *m*ArAT

**Figure 7:**
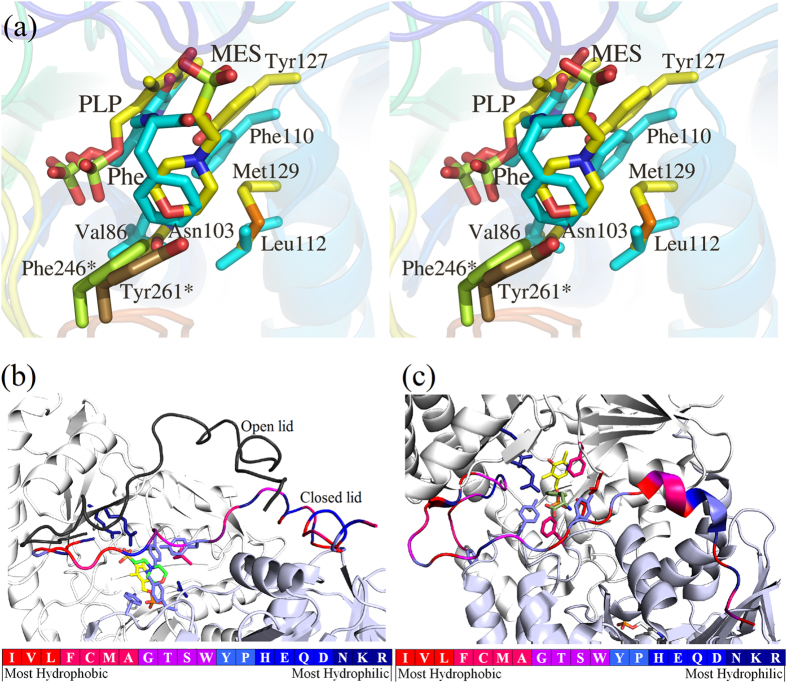
Difference in hydropathicity of residues of *m*HspAT and *m*ArAT active sites. (**a**) A stereoview of the superimposition of the active sites of MES- *m*HspAT and Phe- *m*ArAT complexes shows distinct features. *m*HspAT possesses a water-loving substrate binding pocket (Asn103, Tyr127, Met129 and Tyr261*) and *m*ArAT harbours a hydrophobic one (Val86, Phe110, Leu112 and Phe246*). Hydropathicity character representations of the N-terminal lid and substrate binding region of (**b**) *m*HspAT and (**c**) *m*ArAT. The hydropathicity index^51^ of the individual residues are graphically displayed with the most hydrophobic represented by blue, most hydrophilic represented by red. Substrate binding pockets show a marked difference in the degree of hydropathicity in the two enzymes.

A structural alignment of the MES bound form of *m*HspAT with *e*HspAT bound to PLP and Hsp shows that MES in *m*HspAT binds in a position similar to that of Hsp in *e*HspAT (Fig. 4). In order to chart out the interactions that Hsp utilizes to bind in the active site of *m*HspAT, Hsp was docked into the PLP-*m*HspAT complex. Analysis of the *m*HspAT -MES and *m*HspAT -Hsp structures suggests that the two ligands have similar interactions in the active site pocket (Fig. 6a,c). In particular, three active site residues, Tyr25, Asn103 and Tyr127, are involved in H-bonding with the imidazole ring and amino group of Hsp. Also, Met129 and Pro260 provide van der Waals interactions stabilizing the substrate in the binding pocket. The phosphate moiety of Hsp is held in place by Arg337, Arg346 and Asn176. To determine the molecular basis of substrate specificities of *m*HspAT and *m*ArAT, we compared their active site residues. The chemical environment of their active site pockets, particularly the substrate binding regions, differ markedly (Fig. 7a). *m*HspAT possesses a bowl shaped hydrophilic pocket which is lined by Tyr25, Asn103, Tyr127, Met129, Tyr67* and Tyr261* (* indicates residue protruding from the adjacent protomer). Such a hydrophilic pocket provides a favourable niche for the binding of Hsp and other similar scaffold such as morpholine-ring of MES (Fig. 7b).

Contrastingly in *m*ArAT, residues Val86, Phe110, Leu112 and Phe246* form a hydrophobic substrate binding pocket (Fig. 7c) which provides an energetically favourable environment for the specific binding of hydrophobic substrates such as Phe, Trp and the less polar Tyr. Notably, such a hydrophobic pocket would be unfavourable for the binding of polar substrate such as Hsp, corroborating with the biochemical result that *m*ArAT shows no HspAT activity. These findings suggest that the nature of the residues lining the substrate binding pockets of *m*HspAT and *m*ArAT is the primary determinant of their distinct substrate specificities.

Furthermore, analysis of amino acid residues composition of their N-terminal lids also shows distinct patterns. Like the substrate binding pocket, the N-terminal lid of *m*HspAT comprises of more hydrophilic amino acids, including four Arg, one Lys and three Val. Comparatively, the N-terminus of *m*ArAT is hydrophobic having two Arg, two Lys, four Val and one Ile.

### Structural and sequence similarity of *m*HspAT and *m*ArAT highlights conservation of active site residues

**Figure 8:**
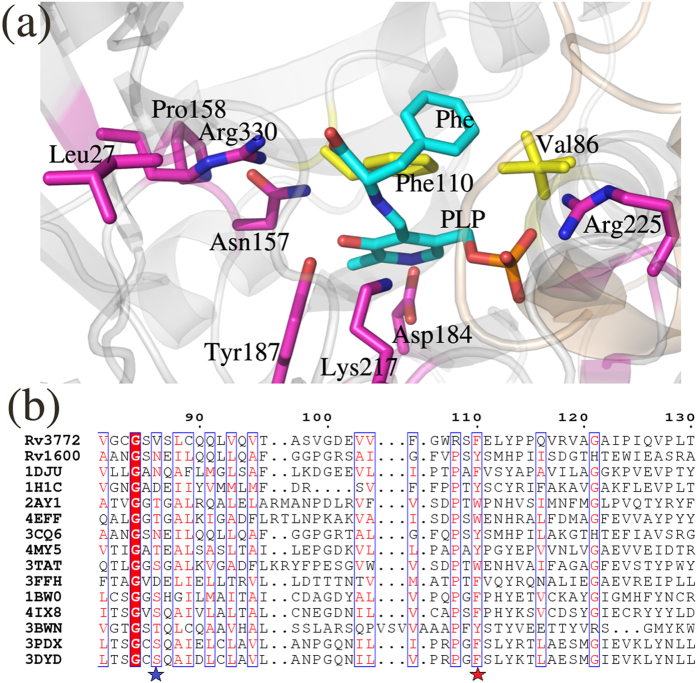
Structural and sequence alignment of selected aminotransferases of subfamily Iβ with *m*HspAT and *m*ArAT. The source of each sequence and PDB ID are: *Pyrococcus horikoshii* ArAT (1DJU)^6^, *T. maritima* HspAT (1H1C)^8^, *Paracoccus denitrificans* ArAT (2AY1)^18^, *Burkholderia pseudomallei* ArAT (4EFF), *C. glutamicum* HspAT (3CQ6)^9^, *Streptococcus mutans* ArAT (4MY5), *E. coli* TyrAT (3TAT)^21^, *Listeria innocua* HspAT (3FFH), *Trypanosoma cruzi* TyrAT (1BW0)^20^, *Leishmania infantum* TyrAT (4IX8)^22^, *Arabidopsis thaliana* TrpAT (3BWN)^23^, Mouse TyrAT (3PDX)^24^ and Human TyrAT (3DYD). (**a**) A close up view of the active site of PLP- *m*ArAT is shown with the strictly conserved residues in magenta colour, whereas the residues which are critical for substrate binding and are partially conserved (0.7 consensus) are shown in yellow. (**b**) A segment of alignment shows that the equivalent positions of Val86 of *m*ArAT is largely occupied by polar residues (marked with a blue star) and equivalent positions of Phe110 of *m*ArAT is occupied by only aromatic amino acids (marked with a red star). The residues that are strictly conserved among the homologous sequences are highlighted in filled-red boxes. Whereas the residue positions showing 70% consensus are highlighted in blue-frame boxes with the similar residues shown in red letters.

To explore the conservation of active site residues of Iβ aminotransferases across species, we performed a structure and sequence based alignment of *m*ArAT and *m*HspAT with homologs from a spectrum of organisms including archae, bacteria, fungi, plants and mammals^6^,^8^,^9^,^18^,^20^,^21^,^22^,^23^,^24^. The residues that are conserved across homologues include those present at positions corresponding to Gly84, Asn157, Pro158, Asp184, Tyr187, Lys217 (forms internal aldimine with PLP), Arg225, Gly227 and Arg330 (involved in anchoring of the phosphate, sulphate and carboxylate moiety of Hsp, MES and Phe, respectively) in *m*ArAT (Fig. 8a). Most of these residues line the active site pocket. The position corresponding to Val86 of *m*ArAT was occupied by hydrophilic residues Ser, Thr, Asp and Asn in the other homologous sequences (Fig. 8b). This suggests that the substrate binding pocket of *m*ArAT is relatively more hydrophobic compared to its counterparts in other organisms. However, the position corresponding to the active site Phe 110 of *m*ArAT is mostly conserved and has an aromatic amino acids in other Iβ homologs (Fig. 8b). The nature of the aromatic amino acid in this position, however, plays a major role in deciding the ligand specificity of the aminotransferase, as is discussed further.

### Inhibition of *m*HspAT and *m*ArAT

Carboxylic acids are known inhibitors of aminotransferases^25^, as they mimic the amino donor and acceptor groups of aminotransferases, *i.e.*, L-glutamate and α-ketoglutarte (α-KG). As seen in the one of the *m*ArAT crystal structure, a dicarboxylic acid, succinate was bound in the active site pocket. Thus, the first choice of molecules for inhibition studies were the dicarboxylate molecules succinic acid and maleic acid, the latter being a well-studied inhibitor of aminotransferases. However, both the dicarboxylates failed to inhibit *m*HspAT and *m*ArAT.

**Figure 9:**
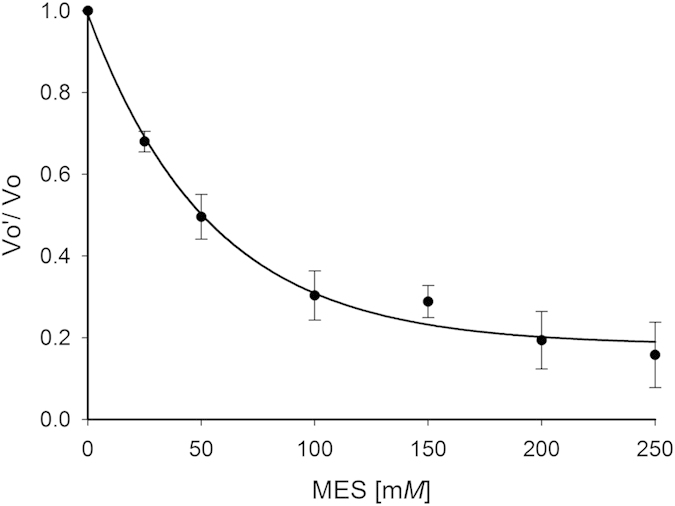
MES specifically, though weakly, inhibits *m*HspAT. Steady state kinetics data using varying concentrations of MES and in the absence of the inhibitor has been plotted, using Hsp as substrate. Vo’ is the velocity of reactions in presence of MES and Vo is the velocity of the uninhibited reaction.

Morphiline-ring compounds such as MES and 4-morpholine propanesulfonic acid (MOPS) have been shown to inhibit enzymes such as metallo-β-lactamase from *Bacteroides fragilis*^26^. We therefore investigated whether the integrally bound MES in *m*HspAT has any inhibitory effect on its enzymatic activity and also, if MES is a specific inhibitor of *m*HspAT. We observed a drastic reduction in the aminotransferase activity of *m*HspAT for Hsp in presence of 50 mM MES (Supplementary Fig. S5a). Further exploration also revealed a concentration dependent inhibition of *m*HspAT-Hsp activity with MES (Fig. 9). However, appreciable inhibition by MES was not seen for the aminotransferase activity of *m*ArAT for Phe (Supplementary Fig. S5b), thereby suggesting a specific, albeit weak inhibitory property of MES. Previously suggested carboxylate derivatives as inhibitors against *Staphylococcus aureus* HspAT^27^ targeted only the phosphate binding residues of PLP and Hsp. We report the first inhibitor which specifically interacts with the hydroxyl group of Tyr127, a residue which is involved in amino-group recognition of Hsp. Thus morphiline-ring based inhibitors may differentiate between enzymes having a Phe in the active site, thereby making this class of molecules a more specific and promising inhibitor of HspATs.

### Mutating a hydrophilic residue to a hydrophobic one affects the recognition of Hsp by *m*HspAT

Taking cue from the structural and inhibition studies, we probed the essentiality of a hydrophilic environment for the binding of Hsp in *m*HspAT. For this, we mutated Tyr127 into a hydrophobic residue, Phe, the equivalent residue in most ArATs. The mutant, Y127F, retained only 1/5th of the activity of the native enzyme for Hsp, irrespective of the concentration of substrate used. However, no significant loss of activity was observed for Phe, the second preferential substrate of *m*HspAT (Supplementary Fig. S6), thus supporting the structural and biochemical results that the interaction of a hydrophilic residue with MES/Hsp is important for *m*HspAT activity.

## Discussion

Worldwide efforts to develop new drugs to combat drug-resistant tuberculosis (TB) have been going on since long. In spite of these efforts, TB continues to infect the world population, causing between 2 to 3 million deaths every year. With the availability of *Mtb* genome sequence in 1998, rational approach for designing anti-TB inhibitors by targeting proteins essential for *Mtb* growth and survival in the host macrophages is gaining momentum. Mounting evidences suggest that many enzymes of the amino acid biosynthesis pathways could be important drug targets for rational design of anti-TB agents^28^. Aminotransferases are one such class of enzymes which are involved in the biosynthesis of a number of metabolites in the cell. The importance of these enzymes is substantiated by the fact that many of them have been targeted for the development of drugs. Examples of human aminotransferases as targets include ornithine aminotransferase for the treatment of hyperammonemias^29^, γ-aminobutyric acid aminotransferase as an anti-epileptic drug^30^ and kynurenine aminotransferase for the treatment of cognitive impairment associated with various psychiatric disorders^31^,^32^. Moreover, a recent study shows that the TyrAT of *Leishmania infantum* is a potential molecular target for the development of anti-leishmanial drug^33^. Thereby, the structural and functional characterization of aminotransferases of important infectious organisms opens new avenues for the development of species specific drugs. Our study on structural and biochemical aspects of two important mycobacterial enzymes *m*HspAT and *m*ArAT is thus relevant for enzyme specific inhibitor design.

Our functional assays clearly showed the inability of *m*ArAT to catalyze Hsp as substrate, but it exhibited broad specificity for aromatic amino acids. *m*HspAT showed high affinity for Hsp and a moderate affinity for the aromatic residues. Crystal structures of *m*HspAT and *m*ArAT showed an overall structural similarity with each other and a structure based sequence alignment of *m*HspAT and *m*ArAT with homologous members of subfamily Iβ also revealed a largely conserved backbone fold. A closer look at the active site architecture of *m*HspAT docked with Hsp unveiled the presence of a tetrad (Asn103, Tyr127, Met129 and Tyr261*) forming a hydrophilic cleft in which an imidazole ring and amino group of Hsp molecule could snugly fit in (Fig. 6c). The substrate binding site of *m*ArAT has a stark difference in its residue composition. It consists of a hydrophobic pocket with an equivalent tetrad being formed by Val86, Phe110, Leu112 and Phe246*. Out of these four residues, Phe110 is mostly conserved in ArATs across species, whereas it is replaced by a Tyr in HspATs (Tyr127 in *m*HspAT). The role of the N-terminal lid has remained unexplored as far as the aminotransferases are concerned. On the basis of findings from the present study, we suggest that this lid plays a crucial role in administering the entry and exit of the substrate. Furthermore, our studies point out to existance of ‘open’ and ‘closed’ structure of *m*HspAT which is defined largely by the movement of the N-termini.

The serendipitous binding of MES to *m*HspAT prompted us to explore its inhibitory property, if any, against *m*HspAT as this enzyme has been proposed as a potential drug target^34^. In addition to being the first report of the inhibitory property of MES for an aminotransferase, the present study also suggests that the binding of the morpholine-ring is specific for *m*HspAT. Therefore, our data lays a foundation to explore MES-like molecules as specific inhibitors of HspATs. Given that amino acids are required in various stages of *Mtb* growth, survival, and defense^35^,^36^,^37^, that many enzymes of amino acid metabolic pathways are potential drug targets^28^ and that humans do not synthesize His, *m*HspAT could be an important target for the design of anti-TB agents. We also compared the closely related human aminotransferases structures to explore their structural similarity to mHspAT. From the structures available in the PDB, except for human kynrenine aminotransferase II, all other aminotransferases showed a significant variation from *m*HspAT in terms of the active site composition. Even the closely related *h*TyrAT showed that its active site is not conducive for the binding of MES (Supplementary Fig. S7). We propose that an MES based scaffold can serve as a platform for developing more potent *Mtb* specific inhibitors, which do not target any of the human aminotransferases.

We also report the experimentally determined structure of *m*ArAT-aromatic amino acid complex. The structural studies on the *m*ArAT suggest that the residues lining the substrate binding pocket dictate preference for aromatic amino acids such as Phe, in addition to the “arginine switch” mechanism proposed in earlier studies^19^. The side chains of hydrophobic residues aid in the binding of Phe in the *m*ArAT and repel polar substrate such as Hsp.

In a nutshell, the structural and functional characterization of the two Iβ aminotransferases from *Mtb* augment the current understanding of His and aromatic amino acid metabolism in *Mtb* and differences in their aminotransferases active sites.

## Materials and Methods

### Enzyme preparation, crystallization and data collection

The details of enzyme preparation, crystallization and preliminary X-ray characterization of both apo *m*HspAT and *m*ArAT in complex with succinate have been reported previously^38^,^39^. Briefly, *rv1600* and *rv3772* were cloned in *M. smegmatis/E. coli* shuttle expression vector pYUB1062 and over-expressed in *M. smegmatis* strain mc^2^4517. The proteins were purified to homogeneity by Ni-NTA affinity and gel filtration chromatography. Apo form of recombinant *m*HspAT was crystallized in PEG MME 2,000, whereas its PLP-complex was prepared by co-purification with PLP (50 μM) in the purification buffer and crystallized in a condition containing MES monohydrate (0.1 M) pH 6.5, ammonium sulphate (0.2 M) and PEG monomethyl ether (MME) 5,000 (30%). *m*ArAT was crystallized in PEG MME 5,000 and the Phe- *m*ArAT complex was obtained by soaking the crystals for 5 min in Phe (2 mM) solution prepared in the mother liquor. X-ray diffraction data from crystals of various forms of both the enzymes were collected using in-house facility as well as synchrotron beam line and were processed using *HKL2000*^40^.

### Structure solution and refinement

The structures of both *m*HspAT and *m*ArAT were solved using the molecular replacement phasing method by the program *PHASER*^41^ of the *CCP4*^42^. The structure of a homologous aminotransferase from *C. glutamicum* (PDB ID: 3CQ5), which shares 59% sequence identity with *m*HspAT, was used as the search model to solve the structure of *m*HspAT. The structure of *m*ArAT was solved using the crystal structure of its *Listeria innocua* counterpart (PDB ID: 3FFH) with which it shares 29% sequence identity. Both the structures were refined in a similar manner using the program *REFMAC5* of *CCP4*. To start with, the model was subjected to 50 cycles of rigid body refinement. Subsequently, 100 cycles of restrained coordinate refinement were carried out using a maximum likelihood target function. At this stage, the *Mtb* specific amino acids were incorporated/substituted into the electron density using the model-building program *COOT*^43^. After every round of model building, positional and isotropic B-factor refinements were carried out. Water molecules were incorporated in the model based on the peak heights (2|F_o_| – |F_c_| at 1σ and |F_o_| –|F_c_| at 3σ contour level) in the electron density maps. In the active site of *m*ArAT, indigenously bound PMP and succinate were modelled based on the Fourier electron density maps. *m*HspAT-PLP-MES complex structure was determined using the same template used for apo *m*HspAT structure determination. The refined coordinates of *m*ArAT-Suc complex was used as the template for determining the Phe bound *m*ArAT structure. The ligand molecules were incorporated into their respective positions on the basis of difference electron density map (|F_o_| – |F_c_|). Subsequently, the complex structures were refined in a manner similar to that employed for the apo structures. The data collection, data processing, and refinement statistics are tabulated in Table 2. The stereochemical acceptability of the structures were validated using the program *PROCHECK*^44^.

### Enzyme kinetics

The aminotransferase activity of *m*HspAT was determined using a two-step assay which involves glutamate dehydrogenase (GDH)^45^ (Supplementary Fig. S1). The assay is based on the transamination of the substrate Hsp or other amino acids in the presence of α-KG resulting in the α-elimination of the amino group. The rate of formation of the 2-oxo acid was monitored spectrophotometrically. The final reaction mixture contained triethanolamine buffer (200 mM, pH 8.4), PLP (0.02 mM), α-KG (2mM), GDH (5 units), NAD (1 mM) and the substrate in varying concentrations. The enzymatic activity was measured at 37 °C by monitoring the reduction of NAD at 340 nm. All reactions were performed in triplicates. Control experiments lacking enzyme or substrate were taken as an estimate of basal level of detection. Glu was excluded from the analysis as it is a by-product of the reaction. Met showed a significant amount of absorbance even in control reaction without the enzyme and hence was excluded from the analysis. The final activities of the enzymes were calculated using a molar extinction coefficient of 6220 M^−1^ cm^−1^ for NADH at 340 nm. For inhibition studies *m*HspAT and *m*ArAT activities were measured in the presence of MES (0–250 mM) with substrates Hsp (1 mM) and Phe (2 mM), respectively.

### Alignment and figure preparation

Sequence and structural alignments were carried out using the programs Clustal Omega^46^ and Align^47^, respectively. Figures were prepared using *PyMOL*^48^, *LigPlot*^49^ and *ESPript* 3.0^50^.

## Additional Information

**Accession numbers**: The atomic coordinates of the models and their corresponding structure factors have been deposited in the Protein Data Bank (www.pdb.org) with the entry codes 4RAE, 4R8D, 4R5Z and 4R2N for apo mHspAT, MES bound PLP-mHspAT complex, and succinate and Phe bound mArAT complexes, respectively.

The corresponding validation reports have been included in the supplemental information.

**How to cite this article**: Nasir, N. *et al.* Crystal structures of *Mycobacterium tuberculosis* HspAT and ArAT reveal structural basis of their distinct substrate specificities. *Sci. Rep.* **6**, 18880; doi: 10.1038/srep18880 (2016).

## Supplementary Material

Supplementary Information

Supplementary Movie S1

## Figures and Tables

**Figure 1:**
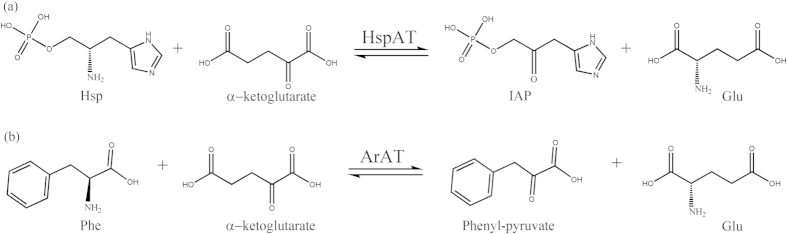
Schematic representation of the biosynthetic steps catalyzed 1β aminotransferases. (**a**) *m*HspAT catalyzes the reversible conversion of Hsp and imidazole-acetol phosphate (IAP) and (**b**) *m*ArAT catalyzes interconversion of Phe and phenylpyruvate. Both the enzymes use PLP as a cofactor and L-glutamate (Glu) as an amino-donor and convert them into PMP and α-ketoglutarate, respectively.

**Table 1:** Kinetic parameters of *Mtb* HspAT and ArAT.

*m*HspAT aminotransferase activity	Hsp	Phe	Tyr
*K*_*M*_[mM]	0.42 ± 0.02	7.1 ± 0.81	9.76 ± 0.69
*k*_*cat*_[s^−1^]	(4.26 ± 0.31) × 10^2^	(2.2 ± 0.13) × 10^2^	(3.1 ± 0.26) × 10^3^
*k*_*cat*_*/K*_*M*_ [M^−1^s^−1^]	(1.02 ± 0.087) × 10^6^	(3.0 ± 0.3) × 10^4^	(3.2 ± 0.35) × 10^5^
*m*ArAT aminotransferase activity	Phe	Tyr	Trp
*K*_*M*_[mM]	0.036 ± 0.001	0.86 ± 0.05	6.26 ± 0.43
*k*_*cat*_[s^−1^]	(3.5 ± 0.14) × 10^2^	(2.78 ± 0.09) × 10^2^	(9.6 ± 0.64) × 10^2^
*k*_*cat*_*/K*_*M*_ [M^−1^s^−1^]	(9.7 ± 0.47) × 10^6^	(3.2 ± 0.21) × 10^5^	(1.54 ± 0.14) × 10^5^

**Table 2:** Data collection and refinement statistics.

*Data collection*	Crystal 1	Crystal 2	Crystal 3	Crystal 4
	Apo-*m*HspAT	*m*HspAT-PLP-MES	*m*ArAT -PMP-Suc	*m*ArAT-PLP-PHE
Space group	P3_2_21	P2_1_2_1_2_1_	P2_1_2_1_2	P2_1_2_1_2_1_
Unit cell dimensions (Å, °)	a = b = 159.94, c = 110.32; α = β = 90, γ = 120	a = 67.52, b = 101.52, c = 114.95; α = β = γ = 90	a = 256.92, b = 77.56, c = 117.91; α = β = γ = 90	a = 55.41, b = 164.46, c = 178.52; α = β = γ = 90
Solvent content (%)	74.5	47.3	66.8	50.0
Temperature (K)	100	100	100	100
Detector	R-AXIS IV^++^	R-AXIS IV^++^	R-AXIS IV^++^	MAR225 CCD
Wavelength (Å)	1.5418	1.5418	1.5418	0.97625
Resolution (Å)	50.00 - 2.60	50.00 - 2.05	50.00 - 1.95	50.00 - 1.98
Highest resolution range	2.69 - 2.60	2.12 - 2.05	2.02 - 1.95	2.05 - 1.98
Unique reflections	47702 (4262)	47586 (3385)	167040 (15927)	113605 (11263)
<I/σ(I)>	9.1 (1.9)	17.1 (1.9)	12.8 (1.9)	15.4 (2.0)
Completeness (%)	94.0 (85.3)	94.6 (68.3)	97.0 (93.6)	100.0 (100.0)
Redundancy	6.3 (3.1)	4.2 (2.2)	5.3 (4.9)	9.6 (8.7)
Rsym (%)^a^	15.9 (44.8)	10.2 (35.1)	14.3 (57.1)	16.9 (94.5)
CC*^b^	0.997 (0.223)	0.998 (0.959)	0.996 (0.990)	0.999 (0.942)
*Refinement*				
Unique reflections (working/test)	45296/2406	45103/2421	158594/8361	107832/5680
R_work_^c^	23.8	20.8	17.9	20.8
R_free_^c^	27.8	25.5	20.3	24.4
*Average B factor (Å*^*2*^)				
All atoms	52.9	38.4	37.9	32.1
Protein atoms	52.9	39.1	29.4	26.5
Ligand	—	35.4	35.5	37.7
Water molecules	—	37.1	37.4	31.4
*R.m.s. deviations from ideal*				
Bond lengths (Å)	0.009	0.008	0.006	0.006
Bond angle (°)	1.4	1.3	1.2	1.1
*Ramachandran plot analysis* (%)				
mostfavored regions	85.3	91.5	90.0	89.3
Additional allowed regions	13.0	8.5	9.4	10.0
Generously allowed regions	1.6	0.0	0.6	0.7
Disallowed regions	0.2	0.0	0.0	0.0

^a^*R*_sym_ (*I*) = ∑_*hkl*_∑_*i*_|*I*_*i*_*(h k l)*– <*I(h k l)* *>* |/∑_*hkl*_∑_*i*_*I*_*i*_*(h k l)* for *n* independent reflectio*n*s and *i* observations of a given reflection. <*I(h k l)*> is the average intensity of the *i* observations. ^b^CC*^52^ was calculated using *PHENIX*^53^. ^c^R_work_ and R_free_ = ∑_h_ ||F(**h**)_o_|–|F(**h**)_c_||/∑_h_ |F(**h**)_o_| where F(**h**)_o_ and F(**h**)_c_ are t**h**e observed and calculated structure-factor amplitudes, respectively. R_free_ was calculated using 5% of data. Values in the parentheses are for the highest resolution range.
