# Supplementary material for: Crystal structures of Mycobacterium tuberculosis HspAT and ArAT reveal structural basis of their distinct substrate specificities
Source: Sci Rep. 2016 Jan 7;6:18880. doi: 10.1038/srep18880 (PMC4703992; doi:10.1038/srep18880)

Supplementary Information

Crystal structures of *Mycobacterium tuberculosis* HspAT and ArAT reveal structural basis of their distinct substrate specificities

Nazia Nasir[1], Avishek Anant[1], Rajan Vyas[1,2] and Bichitra Kumar Biswal[1*]

[1]Protein Crystallography Laboratory, National Institute of Immunology, Aruna Asaf Ali Marg, New Delhi, Delhi, 110067, INDIA

[2]Current address: Department of Physiology and Biophysics, Case Western Reserve University, Cleveland, Ohio, 44106, USA

*To whom correspondence should be addressed: Bichitra Kumar Biswal, Tel.: 91-11-26703705; Fax: 91-11-26742125; Email: bbiswal@nii.res.in

This document includes:

1. Supplementary Tables
2. Supplementary Figures and their Legends
3. Legend of supplementary Movie S1

Supplementary Table S1. A comparison of the Michaelis constant, K_M (mM) of Iβ homologues with their preferred substrates.

Source	*C. glutamicum*	*T. maritima*	*B.subtilis*		*S. typhimurium*
Enzyme	HspAT	HspAT	HspAT	ArAT	HspAT[1]
Substrate					
Hsp	0.8	0.89	0.15	-	20
Phe	38.0	106	2.5	9.8	-
Tyr	2.3	-	0.71	2.7	-
Trp	3.4	-	-	-	-
Leu	-	94.4	-	-	-

1. Albritton, W.L. & Levin, A.P. Some comparative kinetic data on the enzyme imidazoleacetol phosphate:L-glutamate aminotransferase derived from mutant strains of *Salmonella typhimurium*. *J Biol Chem.* **245,** 2525-8 (1970).

Supplementary Table S2. Surface area of interface between two monomers of a homodimer. The values were calculated using *PISA*[1] from the *CCP4* suite using a water probe of 1.4 Å in diameter.

Structure	***Chain ID***	***Buried surface area (Å^2)***
*m*ArAT-succinate	A and B	3125
	C and D	3123
*m*ArAT-Phe	A and C	3291
	B and D	3291
Liganded *m*HspAT	A and B	3524
Unliganded *m*HspAT	A nad B	3447

1. Krissinel, E. & Henrick, K. Inference of macromolecular assemblies from crystalline state. *J Mol Biol.* **372,** 774-797(2007).

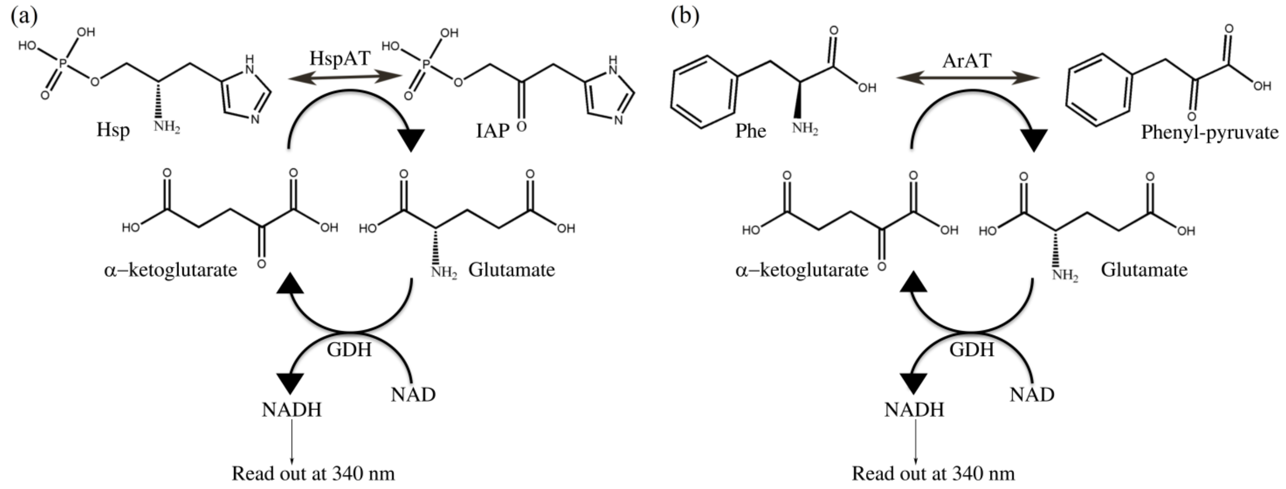

Supplementary Figure S1. **Schematic representation of the GDH-coupled assay system**

The two-step used for determining the activities (a) *m*HspAT and (b) *m*ArAT utilize the same basis.

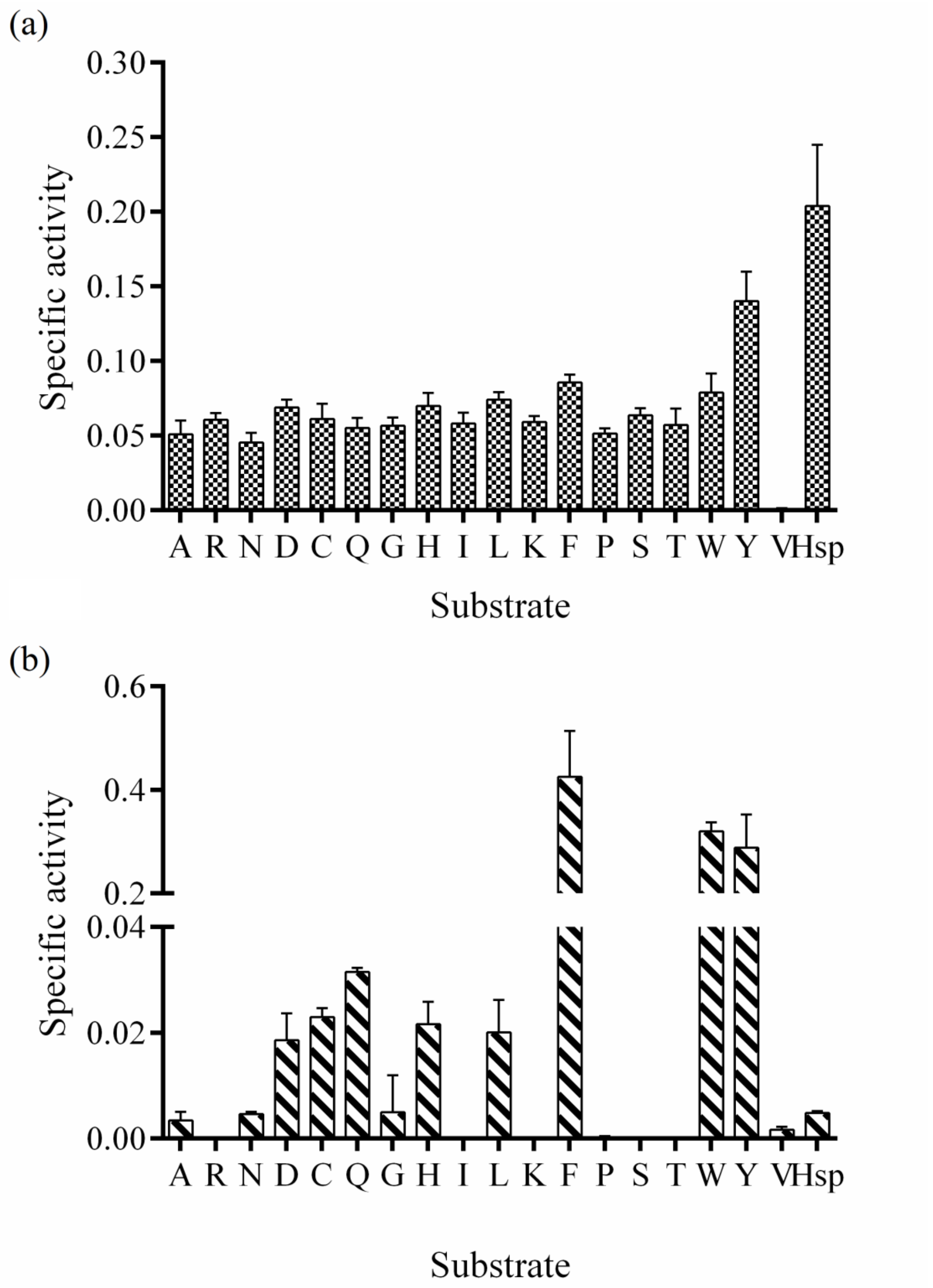

Supplementary Figure S2. **AT activity analysis for *m*HspAT and *m*ArAT using various substrates**.

The specific activity exhibited by the two enzymes for all the amino acids and Hsp as the substrates clearly suggest that (a) *m*HspAT shows maximum activity for Hsp, moderate activity for Trp, Phe and Tyr. (b) *m*ArAT exhibits maximal activity only for aromatic amino acids. The specific activity was calculated in terms of μg of product formed (NADH) per min per mg of enzyme.

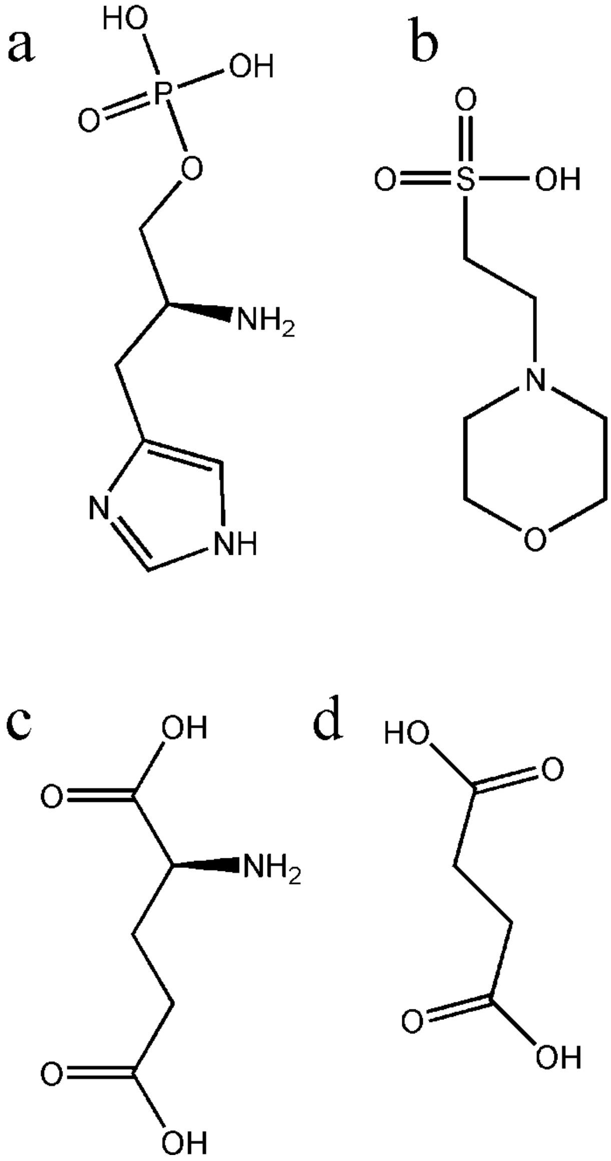

Supplementary Figure S3. Schematic structures of ligands of *m*HspAT and *m*ArAT

The structures include (a) Hsp (b) MES (c) Glu and (d) Succinate.

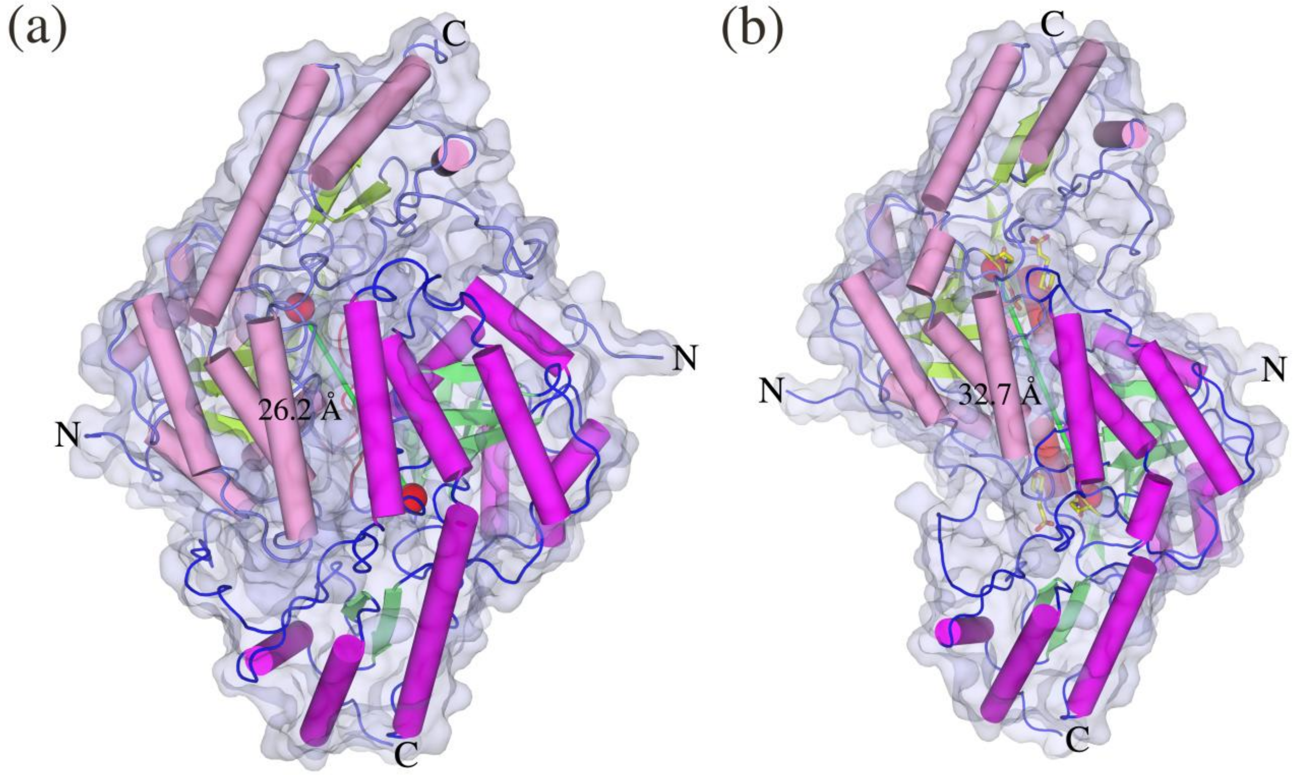

Supplementary Figure S4. **Ligand induced conformational changes at the quaternary structure level of *m*HspAT**.

The dimeric apo form (a) and holo form (b) highlights the difference in the overall dimeric rearrangement upon ligand binding. The apo *m*HspAT dimer adopts a compact structure, as a result of the opening of the N-terminal lid and is clearly demonstrated by the displacement in the CoM (red spheres) by ~7 Å.

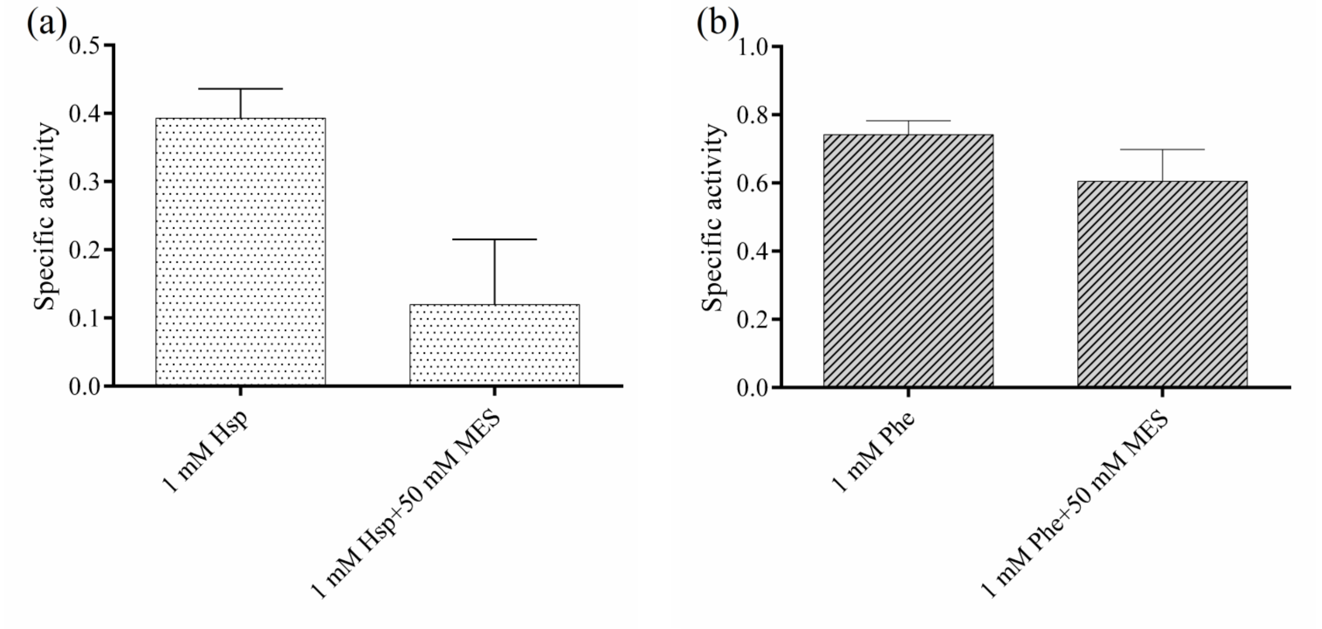

Supplementary Figure S5. MES inhibits *m*HspAT but not *m*ArAT.

(a) Specific activity of (a) native *m*HspAT for Hsp and (b) *m*ArAT for Phe in the absence and presence of 50 m*M* of MES in standard buffer conditions (pH 8.5). The specific activity was calculated in terms of µg of product formed (NADH) per min per mg of enzyme.

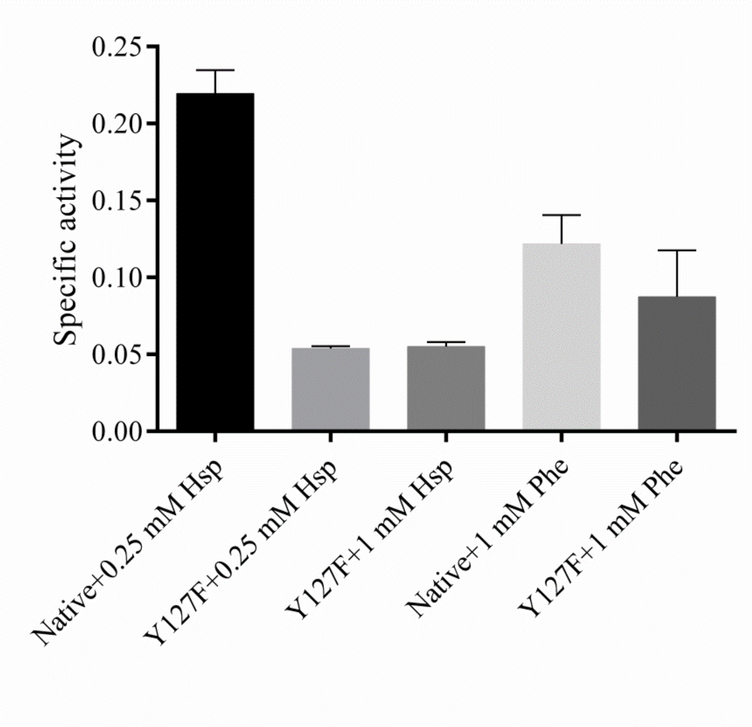

Supplementary Figure S6. Specific activity of *m*HspAT mutant, Y127F.

A comparison of the specific activities of the native enzyme with its mutant for substrates, Hsp and Phe, was performed. A four-fold increase in Hsp concentration (0.25 mM and 1 mM) does not affect the activity of the mutant which retains only about a fifth of that of the native enzyme. This mutant on the other hand retains about the same activity as that of the native enzyme in the presence of 1 mM Phe. The specific activity was calculated in terms of µg of product formed (NADH) per min per mg of enzyme.

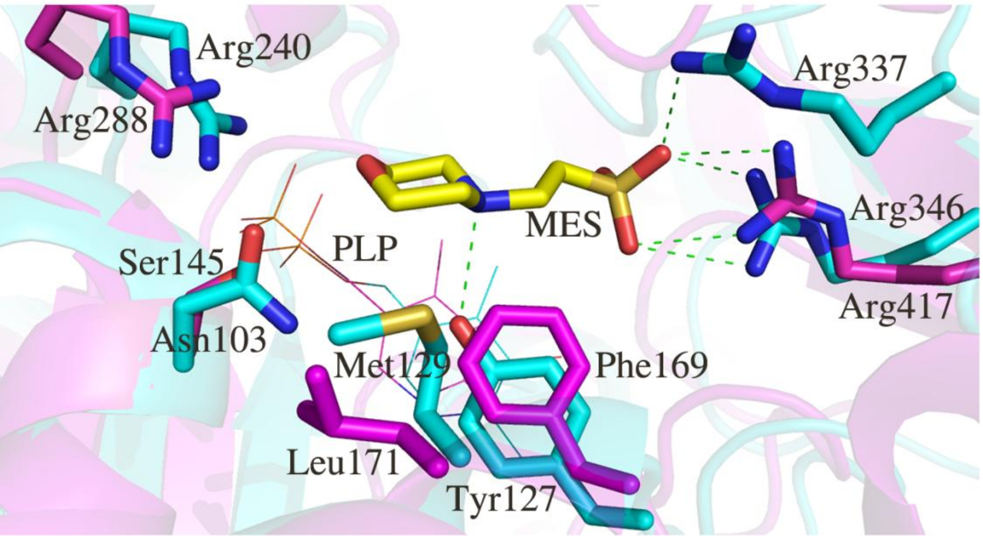

Supplementary Figure S7. Superposition of *h*TyrAT and *m*HspAT active sites

*h*TyrAT is represented in magenta while *m*HspAT is represented in cyan, with MES (from *m*HspAT active site) shown in yellow. The replacement Tyr with Phe and loss of an Arg suggests that MES may not be able to bind *h*TyrAT, possibly making it a specific inhibitor for *Mtb*.

Supplementary Movie S1 (uploaded in .mov format)

The movie depicts the proposed sequence of events involved in the entry of *m*ArAT substrate, Phe and its binding in the active site after replacing the amino-donor mimic, succinic acid. The movie was made using PDBs 4R5Z and 4R2N, *m*ArAT complexed with succinate and Phe, respectively.

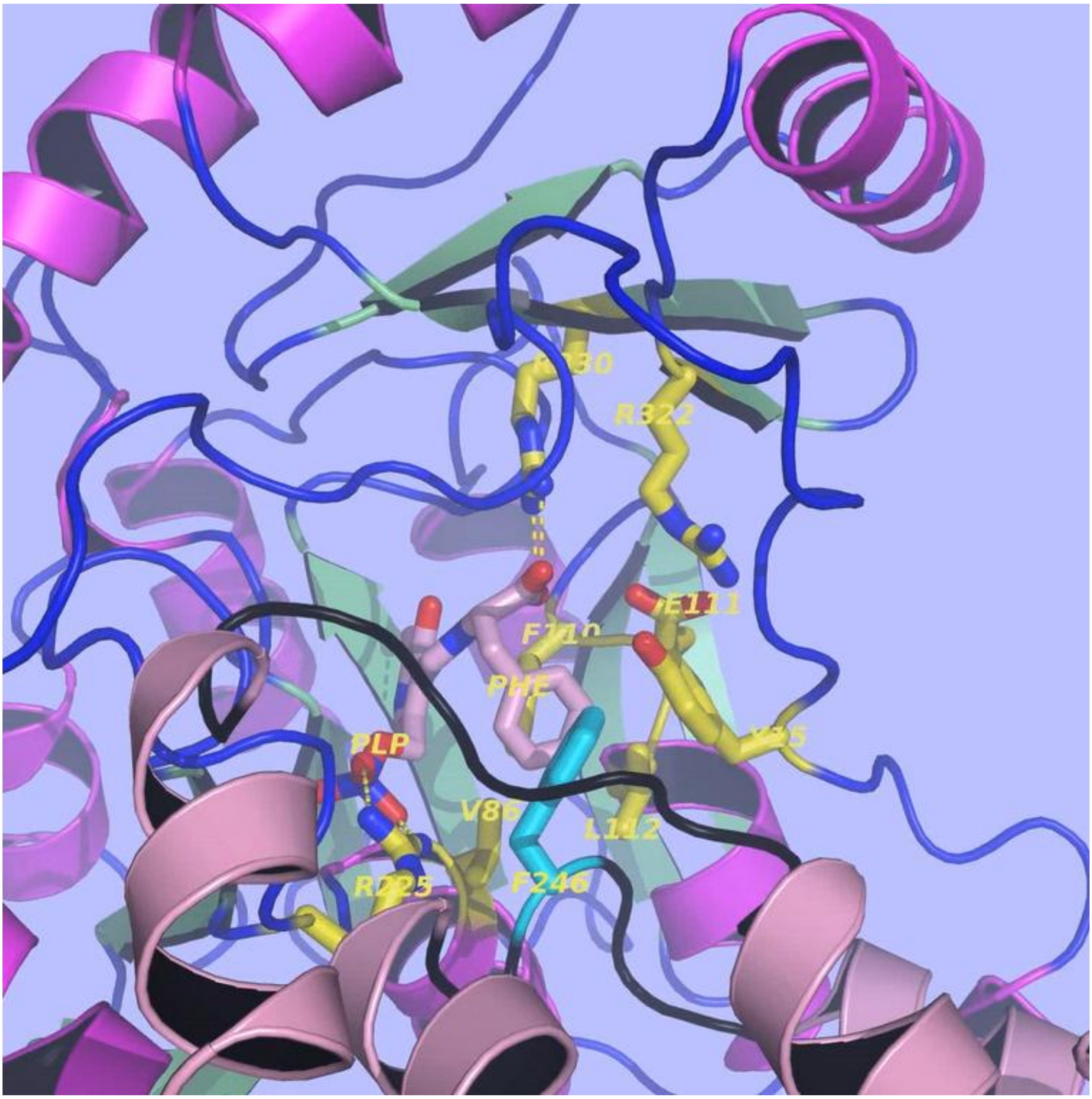

Supplement: Supplementary Information [file srep18880-s1.pdf]
